# Labour intensity of guidelines may have a greater effect on adherence than GPs' workload

**DOI:** 10.1186/1471-2296-10-74

**Published:** 2009-11-28

**Authors:** Michael J van den Berg, Dinny H de Bakker, Peter Spreeuwenberg, Gert P Westert, Jozé CC Braspenning, Jouke van der Zee, Peter P Groenewegen

**Affiliations:** 1 NIVEL, Netherlands Institute for Health Services Research, Utrecht, The Netherlands; 2 Tilburg University, Faculty of Social and Behavioural Sciences, Tilburg, The Netherlands; 3 UMC St Radboud, IQ healthcare, Nijmegen, The Netherlands; 4 Maastricht University, Faculty of Health Sciences, Maastricht, The Netherlands; 5 Utrecht University, Faculty of Social sciences, Utrecht, The Netherlands; 6 RIVM, Netherlands Institute for Public Health and the Environment, Bilthoven, The Netherlands

## Abstract

**Background:**

Physicians' heavy workload is often thought to jeopardise the quality of care and to be a barrier to improving quality. The relationship between these has, however, rarely been investigated. In this study quality of care is defined as care 'in accordance with professional guidelines'. In this study we investigated whether GPs with a higher workload adhere less to guidelines than those with a lower workload and whether guideline recommendations that require a greater time investment are less adhered to than those that can save time.

**Methods:**

Data were used from the Second Dutch National survey of General Practice (DNSGP-2). This nationwide study was carried out between April 2000 and January 2002.

A multilevel logistic-regression analysis was conducted of 170,677 decisions made by GPs, referring to 41 Guideline Adherence Indicators (GAIs), which were derived from 32 different guidelines. Data were used from 130 GPs, working in 83 practices with 98,577 patients. GP-characteristics as well as guideline characteristics were used as independent variables. Measures include workload (number of contacts), hours spent on continuing medical education, satisfaction with available time, practice characteristics and patient characteristics. Outcome measure is an indicator score, which is 1 when a decision is in accordance with professional guidelines or 0 when the decision deviates from guidelines.

**Results:**

On average, 66% of the decisions GPs made were in accordance with guidelines. No relationship was found between the objective workload of GPs and their adherence to guidelines. Subjective workload (measured on a five point scale) was negatively related to guideline adherence (OR = 0.95). After controlling for all other variables, the variation between GPs in adherence to guideline recommendations showed a range of less than 10%.

84% of the variation in guideline adherence was located at the GAI-level. Which means that the differences in adherence levels between guidelines are much larger than differences between GPs. Guideline recommendations that require an extra time investment during the same consultation are significantly less adhered to: (OR = 0.46), while those that can save time have much higher adherence levels: OR = 1.55). Recommendations that reduce the likelihood of a follow-up consultation for the same problem are also more often adhered to compared to those that have no influence on this (OR = 3.13).

**Conclusion:**

No significant relationship was found between the objective workload of GPs and adherence to guidelines. However, guideline recommendations that require an extra time investment are significantly less well adhered to while those that can save time are significantly more often adhered to.

## Background

Physicians' heavy workload is often cited as posing a threat to the quality of care and as a barrier to the implementation of measures to improve quality [1-5]. Although this has often been stated, relatively little effort has been devoted to analysing the relationship between workload and quality of care. In this study we analyse this relationship in a general practice setting. We define workload as the number of consultations handled by GPs within one week. Good quality of care was defined as care in accordance with professional guidelines.

Several studies have cited high workload as a barrier to guideline implementation [6]. However, these studies focus on guideline adherence in general and did not investigate the underlying relationship. Empirical studies on the nature of the relationship between guideline adherence and workload are scarce.

The study of Hutten [1] formed an important first step on this path. However, the data were collected in 1987, when guideline development was still at an early stage. In the past decades the number of professional guidelines has been rising rapidly, so that a better test is possible. More insight into the relationship between workload and guideline adherence can offer valuable information to policy makers and professionals as they strive towards quality improvement.

The purpose of the present study was to investigate the relationship between workload and adherence to professional guidelines. In this we distinguish between the effects of GP workload and the labour intensity of guideline recommendations. We will discuss some theoretical considerations as to why such a relationship is to be expected. This study was carried out in the Netherlands. In the Netherlands guidelines are developed by the Dutch College of General Practitioners. This organisation has a prominent and influential position among GPs. Most Dutch GPs are members of this association and receive all its guidelines and revisions of guidelines. Moreover, the guidelines are published on the internet and are therefore accessible to all who are interested.

### The relationship between stress and job performance

The most plausible assumption appears to be that if workload and guideline adherence are correlated, this correlation will be negative. Workload may be considered to be an indicator for stress due to a lack of time [7]. Psychological research has shown that there is an optimal stress level for workers to perform well [8,9]. A stress level below or above this optimum negatively affects job performance. A number of studies have confirmed the effect of fatigue in clinical settings [10-12].

Consequently, we expect that GPs' workload is negatively related to adherence to professional guidelines.

### Why do some physicians adhere better to guidelines than others?

The acceptance of and adherence to guidelines depends, among other things, on who develops and disseminates them [13,14] and how this is done [15]. The existence of guidelines alone is no guarantee for a change in physicians' behaviour. According to Pathman et al., [16] the process from becoming aware of a guideline to adhering to it, follows four steps: (preawareness)→Awareness→Agreement→Adoption→Adherence. Along this path, the process can be hindered. First, a GP must be aware of the existence of the guideline and familiar with the information contained in it (knowledge). Second, the GP must agree with the guideline and be motivated to implement it (attitude). Indeed, some physicians have negative attitudes towards guidelines in general, because they fear these might promote *'cookbook medicine' *or decrease their autonomy. Third, physicians must, in practice, be able to act in accordance with guidelines; this can be restricted by external barriers [6].

Workload and time pressure are such barriers that negatively affect the first step in the awareness-to-adherence process, because time is needed to stay informed. GPs with a high workload might spend more time on patient care at the expense of time spent on continuing medical education (CME) or reading specialist literature. Accordingly, they might be less informed about the exact content of guidelines. Therefore, we investigated a possible correlation between hours spent on CME and guideline adherence and whether this modifies the relationship between workload and guideline adherence.

According to the theory about the stress-job-performance relationship, this relationship depends on an individual response to 'environmental' events. However, different individuals might perceive and experience the same amount of objective workload differently. Not only will the objective workload be of influence but also the experienced workload. This subjective workload may result in a feeling of being in a rush and not having enough time. This experienced lack of time could be more important than the (objective) amount of available time. Therefore, it is to be expected that experienced high workload also negatively affects guideline adherence and probably modifies the relationship between workload and guideline adherence.

### Why are some guidelines better adhered to than others?

Previous research shows that one of the most important characteristics of a guideline to influence compliance is complexity. Guidelines that are easy to understand, can easily be tried out, and do not require specific resources or skills have a greater chance of being used [17-19,15]. It has also been shown with regard to guidelines about prescriptions, that so-called 'don'ts' are better adhered to than 'dos'. Don'ts are recommendations that say *not *to prescribe something while dos recommend specific drugs [20]. We assume that there is a logical link between the complexity of a guideline and the amount of workload that following this guideline will incur. Since time is scarce for GPs, they will be more likely to adopt guidelines that are simple and less time-consuming. Moreover, GPs with a high workload develop habits and routines to cope with their workload (e.g. spending less time per patient) and might be less likely to change this behaviour even if these routines are in conflict with guidelines. In our study, we also investigate whether guidelines are better adhered to when recommendations are less time-consuming, and whether the negative correlation between workload and guideline adherence is stronger when following the guideline is more time-consuming. Time-consumingness of guidelines was measured as time investment during the same consultation and the chance of return by the patient for the same complaint.

Our research question is: "to what extent is workload an important determinant of guideline adherence?" In this we distinguish between the effects of GP workload and the labour intensity of the guideline recommendations.

## Methods

### Study population

Data were used from the Second Dutch National Survey of General Practice (DNSGP-2) [21]. This nationwide study was carried out between April 2000 and January 2002, in 104 general practices in the Netherlands, comprising 195 GPs and nearly 400,000 listed patients. In each practice, information about patients, contacts, diagnoses, interventions, referrals, prescriptions etc. were recorded during one year. The data of eight practices were excluded from our analyses because they were deemed insufficient. The study was carried out in keeping with Dutch legislation on privacy. Compliance with privacy regulations was approved by the Dutch Data Protection Authority. The methods and data collection of the DNSGP have been described in greater detail by Westert et al [21].

### Data and measurements

**Figure 1:**
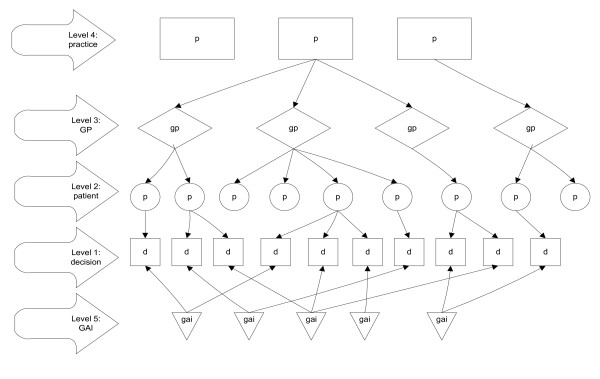
Cross-classified multilevel model with decisions nested in GAIs and in patients, patients in GPs and GPs in practices

The data file used was created by merging several files with data on different levels. This resulted in a dataset with a multilevel structure. The lowest level consists of decisions by GPs, mostly regarding prescriptions or referrals. This is the dependent variable and will be further clarified under 'measures'. These decisions are nested within patients. This means that every decision was made with regard to a patient, and that more decisions can be made concerning the same patient, but that a specific decision never refers to more than one patient. Patients, in turn, are nested within a GP (every GP has more patients, but a patient always has one GP); GPs are nested within practices. The units at the lowest level (decisions) were not only nested within a specific patient, but also within a specific guideline adherence indicator (GAI), which, for instance, indicates that the decision belongs to the indicator 'referring knee complaints to orthopaedist' or 'prescribing antibiotics for sinusitis'. The data structure of this cross-classified model is visualised in figure 1.

We will briefly describe the datasets used in this study. These datasets are also summarised in Table 1:

**Table 1:** Variables used in the analyses: mean/% and standard deviation

	Mean/%	Sd	Type of data source
**Dependent**			
Adherence to guideline	58.7%		Electronic medical records
**Independent**			
***Practice level (n = 83)***			
% publicly insured (per practice)	65.8%	10.1%	Practice administration
% elderly (per practice)	6.0%	2.8%	Practice administration
% ethnic minorities (per practice)	6.3%	11.7%	Patient questionnaire
Nat. logarithm % ethnic minorities	-3.38	1.4	Patient questionnaire
% self-rated health poor (per practice)	19.0%	5.2%	Patient questionnaire
Practice type			National database
Single-handed	57.8%		
Dual practice	20.5%		
Group	12.1%		
Health centre	9.6%		
Urbanization			National database
Very urban	20.5%		
Urban	24.1%		
Suburban	16.8%		
Rural	20.5%		
Very rural	18.1%		
Dispensing practice	10.9%		GP questionnaire 1
***GP-level (n = 130)***			
Workload	114.6	35.4	Electronic medical records
Age	47.4	6.15	National database
Sex (female)	21.5%		National database
List size	2018.3	545.6	Practice administration and GP questionnaire 1
Hours of CME per week	3.2	3.4	Diaries
Satisfaction with available time	2.9	0.7	GP questionnaire 2
			
***Patient level (n = 98,577)***			
Age	39.8	24.0	Practice administration
Sex (female)	56%		Practice administration
Public insurance	71%		Practice administration
Self-rated health poor	18.3%		Patient questionnaire
Self-rated health unknown	22.1%		Patient questionnaire
Non-western ethnic minority	4.6%		Patient questionnaire
Ethnicity unknown	17.8%		Patient questionnaire
			
***GAI level (n = 41)***			
About referrals	32.0%		Electronic medical records
Short-term time investment greater	32.8%		Expert panel
Short-term time investment smaller	22.1%		Expert panel
Long-term time investment greater	19.1%		Expert panel
Long-term time investment smaller	28.6%		Expert panel

#### Electronic medical records

All participating GPs kept electronic medical records. In these records GPs registered the diagnosis using the International Classification of Primary Care (ICPC), and referrals and prescriptions using ATC-codes (Anatomical Therapeutical Chemical classification system).

#### Patient questionnaire

A one-page written questionnaire was sent to all listed patients. This included some characteristics which are not registered in the practice administration, such as self-rated health. The response was 76.5%.

#### Practice administration

The practice administration of all participating practices contains a few items of information on all patients on the practice list: sex, date of birth, insurance status and postal code. There were almost 400,000 patients in the DNSGP-2.

#### GP questionnaires

The GPs received two written questionnaires. The first covered a range of topics about their work. The response to this questionnaire was 96% (188 GPs). The second questionnaire dealt with workload-related issues and job satisfaction. The response to this second questionnaire was 87% (164 GPs).

#### Diaries

The GPs kept a detailed log of their time use for every quarter of an hour in a representative working week. The diary had a pre-structured form with categories such as 'consultation', 'administration', and 'CME'.

#### National database of all GPs

Basic characteristics such as date of birth, sex, single-handed practice or partnership, etc. were retrieved from the national database of GPs [22].

#### Expert panel

Finally, a panel of three practicing general practitioners, working in different practices, was asked to fill out a questionnaire to decide whether a certain decision is associated with a higher or a lower time investment.

All files were merged using unique patient, GP and practice codes for cross-reference between the files. After merging all files, a file with 170,677 records remained, each record representing a decision that was either in accordance with or against a guideline.

All variables used are shown in table 1. In the third column, the type of data source is presented. We will clarify these measures here.

#### Outcome measure: decision in accordance with guideline

Electronic medical records were used for the construction of the dependent variable. This variable is dichotomous and indicates whether a decision is in accordance with the guideline (1) or not (0). This was based on a list of 41 Guideline Adherence Indicators (GAI) which were developed by IQ-healthcare [23,24]. These indicators were based on clinical guidelines developed by the Dutch College of General Practitioners. Each decision refers to an episode, a patient or a contact. The guidelines refer to a specific diagnosis (e.g. acute sore throat). We will illustrate this with an example: A GP notes as diagnosis 'acute sore throat'. The guideline 'Acute sore throat' advises against the use of antibiotics [25]. If the GP prescribed antibiotics during an illness-episode with the diagnosis 'acute sore throat', this decision is coded '0' (against guideline) on our dependent variable. If no antibiotics were prescribed, this is coded as '1' (in accordance with guideline). Obviously, a complete guideline cannot be reduced to one dichotomous variable. Guidelines contain a range of recommendations and considerations that are related to each other and that are often ordered in a decision tree. The GAIs measure specific decisions under certain conditions that play a central role in the guideline and that are relatively simple to measure. The selection of these decisions was done by GPs using an iterative consensus procedure. This method was extensively described elsewhere [23,24,26].

In this way, 213,758 decisions were coded referring to 41 GAIs, mainly about prescribing and referrals. These 41 GAIs were derived from 32 different guidelines. We wanted to be sure that all GAIs referred to situations that happen frequently enough to be relevant and to discriminate between GPs. Therefore, a selection was made on the basis of three criteria:

- the numerator must exceed 100 (in the whole database);

- the indicator must be available for more than 50 practices;

- the denominator divided by the number of practices (which is the average number of times something occurs in one practice) must be higher than 10.

After this selection, 170,677 records (80%) remained.

#### Workload and exactingness of guideline recommendations

- Expected workload effect in actual consultation

The expert panel rated all GAIs on the expected workload in the actual consultation. Every GAI was written as a decision, e.g.: 'prescription of antibiotics to patient with sore throat'. Response categories were 'amount of work in actual consultation is likely to be: greater/equal/smaller'. Some items prescribed a decision that was in accordance with guidelines, other items prescribed a decision that was against the guideline. Answers were recoded into 1, 2 and 3, in such a way that 1 = higher time investment in actual consultation if the guideline is adhered to and 3 = smaller time investment in actual consultation if the guideline is adhered to. All GAIs were given the score on the basis of the majority of the expert ratings (two or three). In the case of three different scores, the GAI was scored as 2. This was the case for one indicator. In 32% there was full agreement between the experts and in 66% two respondents agreed with each other.

- Expected long-term workload effect

This variable was measured in the same way as expected workload effect in actual consultation. The expert panel was asked to rate the likelihood that the patient will return after this decision (greater/equal/smaller). Agreement between the experts was somewhat less. In 10% there was complete agreement, in 68% two experts agreed and in 22% three different ratings were given. The GAIs for which there was no agreement, were scored as 'equal' (2).

- Objective workload of GPs

We measured the workload in terms of the average number of consultations during one week. We extracted these data for one year from the electronic medical records of all listed patients.

- Experienced workload (satisfaction with available time)

This variable is an indicator for subjective workload. In the questionnaire, the GPs filled out a job satisfaction scale originally derived from Cranie et al. [27]. Factor analyses showed that four items formed a scale for satisfaction with available time. This scale consists of the four items: satisfaction with time for family, amount of leisure time, time costs of the practice, available time for CME. Response categories were: very dissatisfied, dissatisfied, partly dissatisfied/partly satisfied, satisfied, very satisfied. The higher the score, the higher the satisfaction with the available time. This scale shows reasonable internal consistency (Cronbach's alpha = 0.78) [28].

- Number of hours per week spent on Continuing Medical Education (CME)

GPs recorded the number of hours spent on CME in the diaries. CME covers doing courses, visiting conferences or reading professional literature.

List size was computed by averaging the number of patients on the list at the beginning of the year and at the end. This list size on a practice level was divided among the GPs within one practice in proportion to their full-time equivalents (FTE). Since a proportion of the GPs work part time, it is important to control for list size. Table 2 shows the correlations between the workload related variables on GP level. Only workload (weekly number of consultations) and list size were significantly correlated: 0.58.

**Table 2:** Bivariate correlations between list size, workload, satisfaction with available time and hours spent on CME

		1	2	3
1	List size			
2	Workload (weekly number of consultations)	0.58**		
3	Satisfaction time	-0.11	-0.07	
4	Hours of CME	0.10	0.05	0.09

* p < 0.05; **p < 0.005

#### Variables at patient level

Since decisions made in clinical practice are also affected by patients, we controlled for five patient characteristics: insurance status, age, sex, self-rated health and ethnicity. We used insurance status, age and sex because these variables are always recorded in the medical file and because they are clearly related to care demand in general. Insurance status was coded as 0 (privately insured) or 1 (publicly insured). Insurance status can be considered as a proxy for social economic status, since until 2006, people above a certain income level were insured privately and people below this income level were insured publicly. Publicly insured people, women and elderly have a significantly higher use of care [29]. Moreover, self-rated health was included because people with low self-rated health will more often suffer from more than one disease and will often have more complicated problems. This can be a reason to deviate from guidelines. Self-rated health was originally measured on a scale from 1 (very good) to 5 (very bad); this was recoded into a dichotomous variable. Scores 1 to 3 were recoded into 0, and scores 4 and 5 (bad and very bad) into 1. Since this variable has many missing values due to non-response, an extra dummy for 'not known' is used in our analyses. Ethnicity was included in the same questionnaire. This was because previous studies have reported ethnic inequalities in the quality of received care [30] and differences in received prescriptions [31]. Moreover, there can be good reasons to deviate from guidelines when ethnic differences are taken into account [32].

#### Background variables at GP level

Age and sex of all participating GPs were collected at the start of the study and were used as controlling variables.

#### Variables at practice level

Dispensing practices, urbanization and practice type were used as controlling variables on practice level. Whether the practice was a dispensing practice was included in the models because many of the GAIs deal with prescriptions. In previous research, it has been shown that GPs in dispensing practices prescribe a broader range of drugs [33]. The degree of urbanization was measured on the basis of the addresses of the practices. There are five categories, varying from very urban to rural. Practice type has four categories: single-handed, dual, group and health centre.

Since the work style of GPs and the presented morbidity might also differ according to case-mix and the composition of the patient population, we added four case-mix variables:

Proportion of publicly insured patients, proportion of elderly (>65+), the proportion of patients with a low self-rated health and the proportion of non-western ethnic minorities. To compute these variables, we aggregated the characteristics of all listed or responding (in case of ethnicity and self-rated health) patients. Since the distribution of ethnic minorities was considerably skewed to the left (indicating that ethnic minorities are highly concentrated within a limited number of practices), this was transformed to a natural logarithm.

#### Controlling variable at GAI-level

Prescription/referral: Most GAIs involve prescriptions or referrals. Only three GAIs are related to other decisions. We coded all GAIs as either 0 (prescription or other) or 1 (referral).

### Statistical Analyses

As explained under 'measures' adherence to 41 separate GAIs was combined within one outcome variable. A score of '1' on this variable means that a GP made a decision that was in accordance with a guideline, a score of '0' means that a GP decided something that was against a guideline. Yet, since we expect that some recommendations are better adhered to than others, we also computed the adherence per GAI. To get an initial impression of the variance in guideline adherence between GAIs and the differences between GPs with a relatively high and a relatively low workload, the proportion of guideline adherence was investigated per GAI and per workload-quartile.

In our multivariable analyses we used a cross-classified logistic multilevel model.

Our dependent variable refers to acting in accordance with guidelines (1) or deviating from guidelines (0). Explanatory variables were added to the model in five steps:

Model 1: workload

Model 2: Workload + background variables of GPs, practices and patients

Model 3: model 2 + hours spent on CME per week and satisfaction with available time

Model 4: model 3 + GAI-characteristics

Model 5: model 4 + interaction terms (workload * workload effect in actual consultation) and (workload * workload effect in long term).

The analyses were carried out in the software programme MLwiN.

## Results

**Figure 2:**
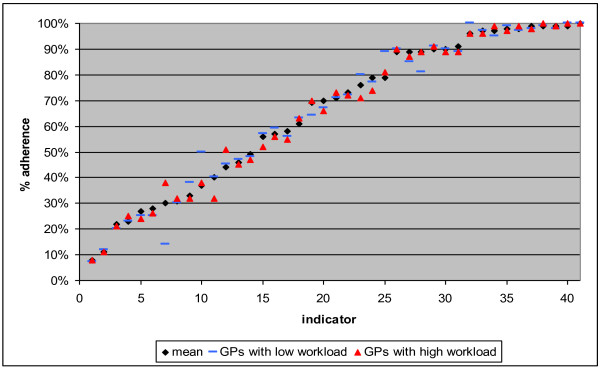
**Proportion of cases in accordance with guidelines, per GAI; mean, GPs with relatively high workload (upper quartile) and GPs with relatively low workload (lowest quartile)**.

Of all decisions in our data, 59% were in accordance with the guidelines. Figure 2 displays the proportion of cases that was in accordance with guidelines per GAI. In the figure, the average proportion of all GPs is shown, the upper workload quartile (GPs with the highest workload) and the lowest workload quartile (GPs with the lowest workload). Clearly, the variation in adherence between the GAIs is large: between 8% and almost 100%. There is also variation between GPs, but this variation is smaller. The variation among GPs differs between GAIs with standard deviations between 1.6 and 39.8. In 44% of the GAIs (18) the adherence was higher among the lowest workload quartile, in 34% (14) the adherence was higher among the highest workload quartile, in 22% there was no difference. Accordingly, no clear correlation between workload and guideline adherence was found.

Table 3 shows the multilevel models. Model 1 shows no correlation between adherence and the GPs' workload. In the other models too, no correlation between objective workload and guideline adherence was found. Likewise, the expectation that the time spent on CME is related to guideline adherence was not confirmed since no significant relationship was found. However, a correlation between subjective workload and adherence was indeed found. In contrast to our expectations this correlation is negative (odds ratio of 0.95). Remarkably, the more satisfied GPs are with their available time, the lower their adherence.

**Table 3:** Multilevel logistic regression analyses of GP-characteristics, practice characteristics, patient characteristics and indicator characteristics on adherence to guidelines

	Model 0	Model 1	Model 2	Model 3	Model 4	Model 5
	**OR**	**OR**	**OR**	**OR**	**OR**	**OR**

Constant	1.893	1.893	1.893	1.066	0.657	0.654
**GP- characteristics**						
Age			1.001	1.002	1.004	1.004
Female GP (ref = male)			1.007	1.021	1.022	1.024
List size			0.952	0.953	0.952	0.947
Workload		1.001	1.000	1.000	1.000	1.000
Hours of CME per week				0.996	0.996	0.996
Satisfaction with available time (1-5)					0.954*	0.954*
**Practice and population characteristics**						
Proportion of elderly 75+			1.024**	1.024**	1.044**	1.045**
Proportion of publicly insured			1.004	1.004	1.007*	1.007*
Proportion of ethnic minorities (Nat. Logarithm)			1.035	1.035	1.067*	1.068*
Proportion of self-rated health poor			0.985*	0.986	0.972**	0.972**
Practice type (ref = single-handed)						
Dual			1.025	1.016	1.027	1.031
Group			1.037	1.015	1.008	1.006
Health centre			1.024	1.010	1.030	1.033
Urbanization (ref = very urban)						
Urban			1.050	1.053	1.017	1.020
Moderately urban			1.102	1.114	1.110	1.111
Rural			1.008	1.006	0.992	0.999
Very rural			1.062	1.075	1.106	1.119
Dispensing practice			0.999	0.984	0.933	0.931
**Patient characteristics**						
Age			1.001**	1.001**	1.002**	1.002**
Female (ref = male)			1.043**	1.043**	1.069**	1.069**
Publ. Insured			1.016	1.015	1.020	1.020
Self-rated health poor			0.967**	0.967**	0.956**	0.956**
Self-rated health unknown			0.962*	0.962*	0.941*	0.941*
Non-western migrant (ref = western)			0.979	0.979	0.956	0.955
Ethnicity unknown			1.030	1.030	1.050*	1.050*
**GAI-characteristics**						
Short-term time investment (ref = equal)						
Greater					0.458**	0.461**
Smaller					1.547**	1.550**
Long-term time investment (ref = equal)						
Greater					2.104**	2.104**
Smaller					3.133**	3.155**
About referrals (ref = prescriptions and other)					15.333**	15.348**
Interaction workload* time investment short greater						0.999
Interaction workload* time investment short smaller						1.000
Interaction workload* time investment long greater						1.000
Interaction workload* time investment long smaller						0.999
						
**Variance components**						
Practice level	0.002	0.001	0.000	0.000	0.00	0.000
Reduction [1]		50%	100%	100%	100%	100%
GP-level	0.005	0.003	0.003	0.003	0.007	0.007
Reduction [1]		32.3%	32.3%	40.2%	0%	0%
Patient level	0.000	0.000	0.000	0.000	0.285	0.287
Reduction [1]						
GAI level	1.965	1.889	1.970	1.971	1.516	1.519
Reduction [1]		4%	0%	0%	22.8%	22.8%

OR = exp-b (odds ratio); *p < 0.05, **p < 0.005

Some strong and statistically significant relationships were found between the required time investment of recommendations and guideline adherence. We expected that recommendations that require an extra time investment during the same consultation, would be less well adhered to. This is supported by our findings. Recommendations that require more time in the same consultation are less adhered to: (OR = 0.46, compared to the 'equal' category). Those that can save time are much better adhered to: (OR = 1.55). Also an expected time investment in the long term is of influence. Recommendations that reduce the likelihood of a follow-up consultation for the same problem are also often adhered to compared to those that have no influence on this (the 'equal' category) (OR = 3.13). Yet, recommendations that increase the chance of a follow-up consultation are also more often followed (OR = 2.10). Recommendations that deal with referrals are significantly more often followed than those concerning prescriptions (OR = 15.35).

No interaction effects were found. This means that the effects found for the GAI recommendations do not differ between GPs with a higher and those with a lower workload. After controlling for all variables, guideline adherence varied between 35.1% and 43.3% among GPs.

**Figure 3:**
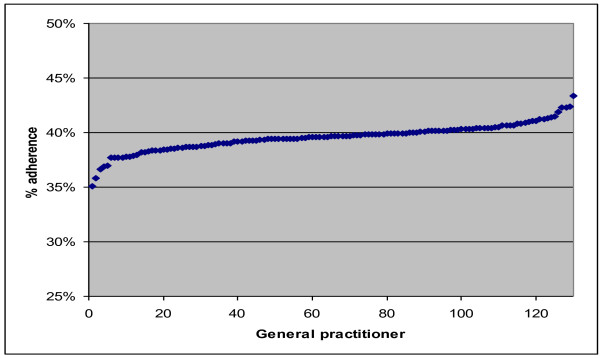
**Proportion adherence to guidelines per GP, after correction for all variables**.

At the bottom of table 3 the variance components are shown; over 99% (1.965) of the higher level variance was located at the GAI level. After adding the other variables, some shifts took place between the different components. In our final model, still 84% of the variance was located at the GAI level; the remaining part was located at the patient level. Figure 3 shows the percentage of adherence, per GP, after controlling for all other variables. As we can see, the differences are relatively small: a range of less than 10% between the extremes. Note that the scores in the figure are estimated on the basis that all other variables equal 0. For most variables this was the average score, but also the variables 'about referrals' have a value of 0 in the equation, which means that the score is estimated on the basis that the decision is not related to referrals.

## Discussion and conclusion

The main question in this study was whether there is a relationship between workload and guideline adherence. We did not find any differences in guideline adherence between GPs with a higher and those with a lower objective workload. However, we found marked differences between guideline recommendations that require a time investment and those that require no extra time. Recommendations that require an extra time investment were less well adhered to.

The expectation that the time spent on keeping up to date influences guideline adherence was not confirmed. Again, this is in line with previous results (Hutten [1]). A possible explanation for the absence of this relationship is that the Netherlands has a mandatory credit points based system for CME. A minimum of 40 hours per year is required to retain registration as a GP. Besides, the recommendations in the guidelines are clearly described, easily accessible and mostly deal with frequently occurring complaints.

We did observe a small but statistically significant relationship between experienced lack of time (subjective workload) and guideline adherence. However, the finding runs contrary to our expectation: higher satisfaction with available time is found to be correlated to lower guideline adherence. Zantinge et al. [34] found that GPs who experience a lack of time are less patient-centred. This could possibly lead to a tendency to fall back on guidelines and to provide more 'standard' care. The relationship is, however, very small. A better understanding of this relationship requires further investigation.

The relationship that we found between short-term time investment and adherence is in line with our expectation: recommendations that require more time investment are followed significantly less often; those that reduce the time investment are more often followed. These correlations are quite strong and are statistically significant. We also found that recommendations that are less likely to induce follow-up consultations are more often adhered to. Contrary to our expectation, recommendations that are likely to lead to follow-up consultations are likewise more often followed compared to the 'equal' category. Of course, the GP's choice whether or not to follow guidelines is constrained by medical considerations. Workload is only one factor in the decision process and despite their workload, GPs are obviously concerned for the wellbeing of their patients. This probably explains why recommendations that incur follow-up consultations are better adhered to.

Two important methodological considerations will be discussed here. First, we want to underline the importance of the cross-classified modelling we used. If we had not included the GAI-level, we would have concluded that some GPs have a higher adherence rate than others, without noticing that this is due to the simple fact that some GPs have a higher number of contacts that are related to GAIs that are better followed in general. We checked this by repeating the analyses without including GAI-level, which resulted in a considerable variation between GPs.

Second, in the literature about guideline adherence, sometimes a distinction is made between so-called 'dos' and 'don'ts'; recommendations that advise to do something and those that advise *not *to do something. It may appear obvious that doing something will generate more workload than *not *doing something and thus, that our expert panel rated the dos as more burdensome than the don'ts. This was, however not the case. Prescribing, for instance, often generates less workload than explaining why the patient does not get a prescription. There was no clear relationship between the expected workload and whether the recommendation was a 'do' or a 'don't'.

Some remarks will be made about the limitations of this study. First, it should be noted that guideline adherence is only a part of the quality of care. Many aspects of quality, such as communication style and organisation are beyond the scope of this study. There is no one-on-one relationship between guideline adherence and quality. In some cases, there are good reasons to deviate from guidelines. These reasons will often be related to patients or to morbidity, but not to GPs and practices. Previous studies have shown that comorbidity can be a reason to deviate from guidelines [15]. This factor was not controlled for in this study. It is, however, unlikely that comorbid conditions will vary strongly between GPs or practices after controlling for age and self-rated health. Second, our data contain only cases that could be measured by an indicator. The content of the guidelines encompasses many more recommendations that were not measured, due to the simple fact that not all GPs actions are recorded in a file. Third, in our analyses, workload was considered a stable characteristic at individual GP level, i.e. some GPs are consistently busier than others. At the same time, workload can also vary between days. Consequently, it seems plausible that the same GP might make other decisions on busy days than on less busy days. To determine how busy a GP was on a specific day, one needs the number of contacts on that day as a numerator and the number of working hours as a denominator. The latter was, however, not known. Fourth, the data used in this study are relatively old. It was, however the most recent database available with this specific information. When more recent data are available, it should be investigated whether the relations that we found have been changing over time. Fifth, there are possibly factors that were not included in our analyses but do influence adherence. These might be individual preferences of patients or specific conditions in the situation of patients that can not be derived from electronic records.

The finding that the required time investment incurred by a recommendation was strongly correlated with adherence, in combination with the fact that an overwhelming proportion of the variance was located on the GAI level, leads to two important conclusions. First, in the Netherlands, adherence to guidelines seems to depend on the content of the guidelines to a far greater extent than on the GPs. As described in the introduction, a great effort has already been made in the Netherlands to promote and disseminate the guidelines. It is therefore likely that in countries where guidelines have a less firm position, more variation between GPs will be found and that thus, there is more to gain by encouraging GPs to adhere to and to adopt guidelines. Second, when developing guidelines, it seems sensible to take the required time investment of recommendations into account, since this may affect the likelihood that recommendations are followed.

## Competing interests

The authors declare that they have no competing interests.

## Authors' contributions

MB was involved in the original idea, design, analysis and interpretation of the data and wrote the manuscript. DB was involved in the data collection and the design of the DNSGP and contributed to the interpretation of the data and the critical revision of the manuscript. PS contributed to the statistical analyses and interpretation of the data. GPW was involved in the data collection and the design of the DNSGP, the design of the study and contributed to the critical revision of the manuscript. JCCB directed the development of all quality indicators used in this study and contributed to the critical revision of the manuscript. JZ was involved in the data collection and the design of the DNSGP and contributed to the critical revision of the manuscript. PPG was involved in the original idea, design, analysis and interpretation of the data and contributed to the critical revision of the manuscript. All authors read and approved the final manuscript.

## Pre-publication history

The pre-publication history for this paper can be accessed here:

http://www.biomedcentral.com/1471-2296/10/74/prepub
